# Liquid Chromatography Tandem Mass Spectrometry Quantification of ^13^C-Labeling in Sugars

**DOI:** 10.3390/metabo10010030

**Published:** 2020-01-10

**Authors:** Jean-Christophe Cocuron, Zacchary Ross, Ana P. Alonso

**Affiliations:** 1 BioDiscovery Institute, University of North Texas, Denton, TX 76203, USA; 2 Heritage College of Osteopathic Medicine, Ohio University, Dublin, OH 43016, USA; 3 Department of Biological Sciences, University of North Texas, Denton, TX 76203, USA

**Keywords:** sucrose, glucose 6-phosphate, fructose 6-phosphate, subcellular compartmentation, ^13^C-labeling, LC-MS/MS, hexokinase, invertase, ^13^C-metabolic flux analysis

## Abstract

Subcellular compartmentation has been challenging in plant ^13^C-metabolic flux analysis. Indeed, plant cells are highly compartmented: they contain vacuoles and plastids in addition to the regular organelles found in other eukaryotes. The distinction of reactions between compartments is possible when metabolites are synthesized in a particular compartment or by a unique pathway. Sucrose is an example of such a metabolite: it is specifically produced in the cytosol from glucose 6-phosphate (G6P) and fructose 6-phosphate (F6P). Therefore, determining the ^13^C-labeling in the fructosyl and glucosyl moieties of sucrose directly informs about the labeling of cytosolic F6P and G6P, respectively. To date, the most commonly used method to monitor sucrose labeling is by nuclear magnetic resonance, which requires substantial amounts of biological sample. This study describes a new methodology that accurately measures the labeling in free sugars using liquid chromatography tandem mass spectrometry (LC-MS/MS). For this purpose, maize embryos were pulsed with [U-^13^C]-fructose, intracellular sugars were extracted, and their time-course labeling was analyzed by LC-MS/MS. Additionally, extracts were enzymatically treated with hexokinase to remove the soluble hexoses, and then invertase to cleave sucrose into fructose and glucose. Finally, the labeling in the glucosyl and fructosyl moieties of sucrose was determined by LC-MS/MS.

## 1. Introduction

Metabolic flux is the flow of matter through a metabolic step or pathway. The goal of ^13^C-metabolic flux analysis (MFA) is to quantify all the in vivo intracellular metabolic fluxes in a given organ or cell, which results in a metabolic flux map. While only a few fluxes, such as the rates of substrate uptake and product accumulation can be measured directly, determination of intermediary carbon fluxes requires ^13^C-labeling. Labeled plant tissues/cells are usually harvested after they have reached isotopic steady-state, meaning that the labeling in intermediary metabolites and products have reached a constant pattern. The resultant labeling in a range of metabolites is then determined using nuclear magnetic resonance (NMR), which gives positional isotopomers information, while mass spectrometry (MS) is used to provide mass isotopomer information [1].

First MFA studies considered cells as a unique compartment where organelles were not taken into account [2,3,4]. Although the importance of subcellular compartmentation in the regulation of metabolism is well established [5,6], its application in MFA—especially in plants—has been challenging. Indeed, plant cells are highly compartmented: in addition to the regular organelles found in other eukaryotes, they contain vacuoles and plastids. Consequently, different pools of the same metabolite can be present in different compartments, and some reactions and even entire pathways are duplicated, which adds to the complexity of applying MFA to plant cells [1,7]. For instance, glucose 6-phosphate (G6P), one of the intermediaries of the glycolysis, is commonly found in the cytosol where it serves as a precursor for sucrose and cell wall biosynthesis, and in the plastid where it is involved in starch production. Similarly, the full glycolytic pathway is usually redundant between the cytosol and the plastid. Recent ion chromatography tandem mass spectrometry techniques have been developed to directly monitor the labeling of various glycolytic intermediaries, such as G6P [8,9]. However, the metabolite extraction procedure is pooling together compounds present in different organelles, which results in an average labeling for each intermediary and hence a loss of subcellular information.

**Figure 1:**
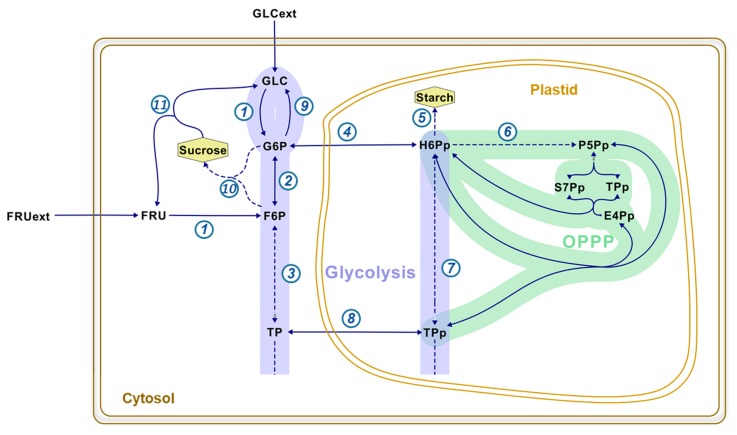
Representative example of maize embryos to illustrate the subcellular compartmentation of hexose-phosphates in plant cells. Developing maize embryos mainly import glucose and fructose as carbon sources. These sugars get phosphorylated into hexose-phosphate in the cytosol. The resulting hexose-phosphates are involved in major pathways: glycolysis (purple), and the oxidative pentose-phosphate pathway (OPPP; green). Full and dashed arrows represent one reaction or a multiple-step reaction, respectively: 1 is hexokinase; 2, phosphoglucose isomerase; 3 and 7, cytosolic and plastidial aldolases, respectively; 4 and 8, exchange of hexose-phosphates and triose-phosphates between cytosol and plastid, respectively; 5, starch synthesis; 6, oxidative part of the OPPP; 9, glucose 6-phosphatase; 10, sucrose phosphate synthase; and 11, invertase. Abbreviations in alphabetical order: E4Pp, plastidic erythrose 4-phosphate, FRU(ext), (extracellular) fructose; F6P, fructose 6-phosphate; GLC(ext), (extracellular) glucose; G6P, glucose 6-phosphate; H6Pp, plastidic hexose-phosphates; P5Pp, plastidic pentose-phosphates; S7Pp, plastidic sedoheptulose 7-phosphate; TP(p), (plastidic) triose-phosphates.

The distinction of reactions between compartments is possible when metabolites are synthesized in a particular compartment or by a unique pathway [10]. For instance, starch is exclusively produced in the plastid, and its labeling pattern is a direct reading of the labeling in plastidic hexose-phosphates (Figure 1). Sucrose is another example of such a metabolite: it is specifically synthesized in the cytosol from G6P and fructose 6-phosphate (F6P) through the activity of the sucrose phosphate synthase (#10, Figure 1). Therefore, determining the ^13^C-labeling in the fructosyl and glucosyl moieties of sucrose directly informs about the labeling of cytosolic F6P and G6P, respectively. The capability of distinguishing between the hexose-phosphates from the cytosol and the plastid has been crucial to determine: (i) the subcellular location of the oxidative pentose phosphate pathway; (ii) the exchange rates of hexose-phosphates between these two compartments; and (iii) the portion of the glycolytic flux occurring in the cytosol vs. the plastid (Figure 1) [11,12,13,14,15,16,17,18,19]. Additionally, the analysis of the labeling of the free sugars also lead to the discovery of a cytosolic cycle re-synthesizing glucose from G6P through the synthesis and degradation of sucrose and/or the activity of a potential glucose 6-phosphatase [11,12,13,14,15,16,17,19,20]. Substrate cycles are also called “futile cycles” because they consume ATP without apparent physiological function [21]. Indeed, these processes can be high-energy demanding and the overall ATP consumption varies from 5% to 70%, depending on the plant organ [2,12,13,17,19,20].

Although a recent study used high-resolution mass spectrometry to determine the labeling of free sugars (glucose, fructose, and sucrose) in plant cells, it does not provide information on the glucosyl and fructosyl units from sucrose [22]. To date, the most commonly used method to monitor the labeling in sucrose fructosyl and glycosyl moieties is by NMR [11,12,13,14,15,16,17,18,19,23]. Although NMR provides positional labeling information, its sensitivity is limited in comparison to MS, consequently requiring substantial amounts of biological sample. This study describes a new methodology that accurately measures the labeling in free sugars using liquid chromatography tandem mass spectrometry (LC-MS/MS) in multiple reaction monitoring mode. This approach was applied to monitor the time-course labeling of glucose, fructose, sucrose and its glucosyl and fructosyl moieties in maize embryos incubated with [U-^13^C]-fructose.

## 2. Results and Discussion

### 2.1. LC-MS/MS Analysis of Free Sugars

Maize ears were harvested at 18 days after pollination (DAP), and embryos were dissected from the kernels, lyophilized and ground into a fine powder. Free sugars were extracted using boiling water, and analyzed by LC-MS/MS as previously described [16,24]. Briefly, sugars were separated using a gradient of acetonitrile and water on a Shodex Asahipak NH2P-50 2D column (2.0 × 150 mm) and a Shodex Asahipak NH2P-50G 2A guard column. The detection was performed by a triple-quadrupole QTRAP 5500 from AB Sciex in negative mode using multiple reaction monitoring (MRM) and parameters listed in Table 1. Hexoses and disaccharides were followed using transitions 179/89 and 341/59, respectively, which correspond to [parent]/[daughter] ion formulas of [C_6_H_11_O_6_^−^]/[C_3_H_6_O_3_^−^] and [C_12_H_21_O_11_^−^]/[C_2_H_4_O_2_^−^] [25]. This methodology was demonstrated to successfully separate sugar isomers: (i) fructose, mannose, galactose, glucose, and inositol, and (ii) sucrose, maltose, and trehalose [24].

**Figure 2:**
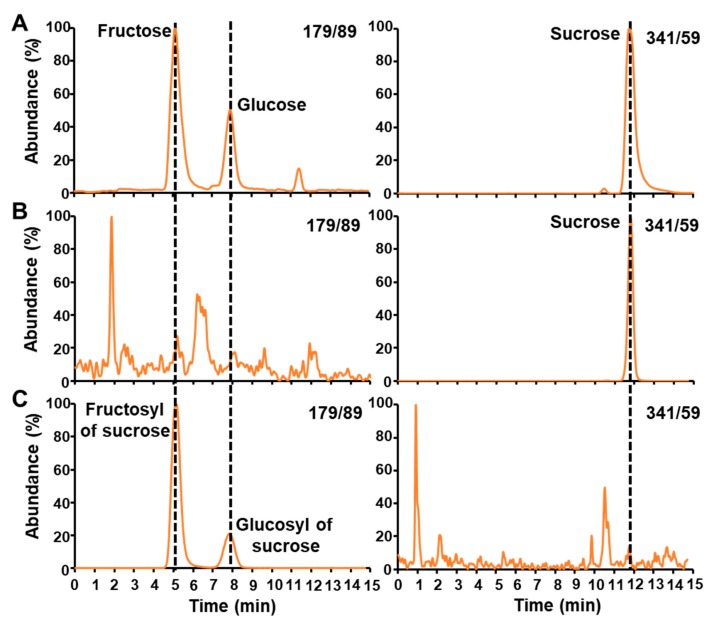
LC-MS/MS chromatograms of free sugars before and after enzymatic treatments. (**A**) shows the chromatographic separation and mass spectrometric detection of fructose, glucose (transition 179/89), and sucrose (transition 341/59) after boiling water extraction of unlabeled maize embryos. (**B**) and (**C**) represent the LC-MS/MS chromatograms obtained after hexokinase and invertase treatments, respectively. LC-MS/MS chromatograms for hexoses and sucrose were obtained using MRM scan survey approach as indicated in the Materials and Methods.

Three main sugars were found in maize embryos: (i) fructose was followed in the transition of the hexoses (179/89) with a retention time of 5.11 min; (ii) glucose, another hexose (transition 179/89), comes at 7.90 min; and (iii) sucrose was monitored in the transition of the disaccharides (341/59) and has a retention time of 11.95 min (Figure 2A).

### 2.2. LC-MS/MS Analysis of the Glucosyl and Fructosyl Moieties of Sucrose

Boiling water extractions from maize embryos were treated first with a hexokinase from *Saccharomyces cerevisiae* for two hours in order to phosphorylate the free glucose and fructose into G6P and F6P, respectively. As shown in Figure 2B, the hexokinase entirely converts all the hexoses present in maize embryos. Indeed, no peaks of glucose and fructose were detected at their expected retention time after this operation. It is important to note that the sucrose was left intact (Figure 2B). Then, an invertase from *Saccharomyces cerevisiae* was added for two hours in order to cleave the sucrose into its fructosyl and glucosyl moieties. The resultant sugars units were analyzed by LC-MS/MS (Figure 2C). Invertase cleaved all the sucrose, which is denoted by the absence of a peak at its expected retention time. The fact that no sucrose was detected after invertase treatment indicates that it was entirely converted into glucose and fructose. Indeed, two peaks appears in the transition of the hexoses (179/89) corresponding to the fructosyl and glucosyl of sucrose.

Sucrose is an important metabolite in terms of subcellular localization: it is specifically synthesized in the cytosol from G6P and F6P by the activity of the sucrose phosphate synthase (#10, Figure 1). The enzymatic steps described above will give access to the ^13^C-labeling in the fructosyl and glucosyl moieties of sucrose, which will directly inform about the labeling of cytosolic F6P and G6P, respectively.

### 2.3. Application of LC-MS/MS to Monitor ^13^C-Labeling in Free Sugars

Previous labeling experiments consisted in incubating maize embryos from 15 to 21 DAP with ^13^C-labeled glucose until isotopic steady state, and revealed the occurrence of cycle of re-synthesis of glucose from G6P [11,16]. However, steady state labeling does not allow the distinction between the sucrose substrate cycle and the glucose 6-phosphatase activity because both reactions result in the same labeling distribution. To discern between the two potential reactions, a pulse labeling experiments is required. For this purpose, maize embryos were harvested at 18 DAP (corresponding to the half-time of the previous steady state labeling experiments), and pulsed with [U-^13^C] fructose for 1, 2, 5, 20, 80, 320, 1280, 2730, 4160, and 5600 min while the other carbon sources in the medium were ^12^C-glucose and ^12^C-glutamine. We strategically chose to use [U-^13^C] fructose, and to monitor the appearance of the labeling in free intracellular glucose as an additional proof that these substrate cycles are occurring in plant cells. Intracellular sugars were extracted from labeled maize embryos using boiling water. The time-course labeling of free glucose, fructose, and sucrose was monitored by LC-MS/MS using multiple reaction monitoring mode and MS parameters listed in Table 1 and Table 2.

**Figure 3:**
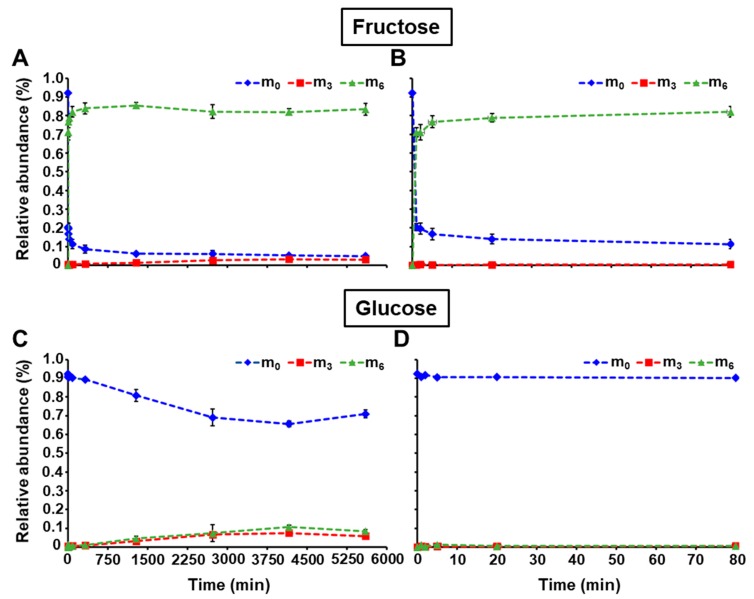
Relative abundance of isotopomers for intracellular fructose and glucose during pulse labeling with [U-^13^C]fructose. (**A**,**C**) show the relative abundance of mass isotopomers at all time points for fructose and glucose, respectively. (**B**,**D**) represent the relative abundance of mass isotopomers from 0 to 80 min for fructose and glucose, respectively. Intracellular glucose and fructose were extracted, and analyzed as described in the “Materials and Methods” section. Mass relative abundances (in %) were reported as the average ± SD (*n* = 4 biological replicates). Mass isotopomers m_0_, m_3_, and m_6_ are depicted as blue diamond, red square, and green triangle, respectively.

The ^13^C-isotopomer abundances of fructose and glucose are reported in Table S1. Figure 3 depicts the relative abundances of the isotopomers m_0_ (unlabeled hexose), m_3_ (labeled in three carbons), and m_6_ (fully labeled) over the entire time course, and the first 80 min. The uptake of [U-^13^C]-fructose from the medium is quickly labeling the intracellular fructose whose m_0_ rapidly decreased to 16.7% within the first 5 min, and then slowly reached a value of 4.9% at 5600 min. On the other hand, the fully labeled fructose (m_6_) reached 76.7% the first 5 min of the pulse, and continued to steadily increase to 83.4% at the end of the incubation. Interestingly, the appearance of other mass isotopomers, such as m_3_, was also monitored and will be discussed below (Figure 3, Table S1 (Supplementary Materials)). The labeling profile was found to be much slower for intracellular glucose due to the uptake of unlabeled glucose from the medium. Indeed, the occurrence of m_3_ and m_6_ only started after 320 min of incubation and their relative abundance reached 5.9% and 8.5%, respectively (Figure 3, Table S1 (Supplementary Materials)).

**Figure 4:**
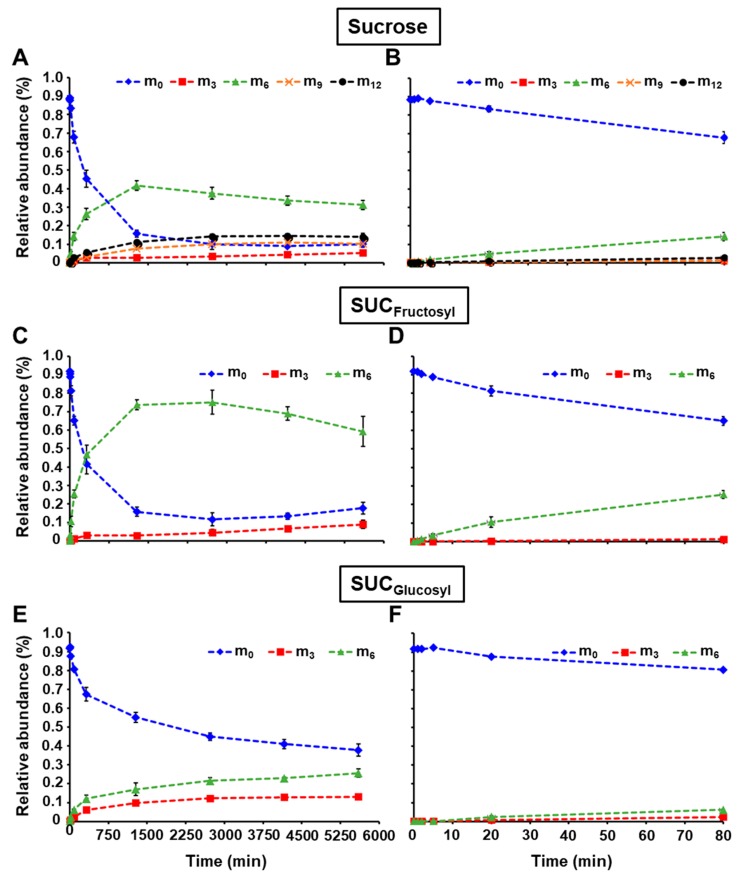
Relative abundance of isotopomers for ^13^C-fructosyl and ^13^C-glucosyl units from sucrose during the pulse labeling experiment. (**A**,**C**,**E**) represent the relative abundance of mass isotopomers at all time points for sucrose, SUC_Fructosyl_, and SUC_Glucosyl_, respectively. (**B**,**D**,**F**) depict the relative abundance of mass isotopomers from 0 to 80 min for sucrose, SUC_Fructosyl_, and SUC_Glucosyl_, respectively. Sucrose was extracted, cleaved into its fructosyl and glucosyl units, and analyzed as described in the Materials and Methods section. Mass relative abundances (in %) were reported as the average ± SD (n = 4 biological replicates). Mass isotopomers m_0_, m_3_, m_6_, m_9_, m_12_ are shown as blue diamond, red square, green triangle, orange cross, and black circle, respectively. SUC_Fructosyl_: Sucrose fructosyl; SUC_Glucosyl_: Sucrose glucosyl.

The relative abundance of intracellular sucrose mass isotopomers is detailed in Table S2 (Supplementary Materials), and plotted in Figure 4A,B. Additionally, maize embryo extracts were enzymatically treated with hexokinase to remove the soluble hexoses, and then invertase to cleave sucrose into fructose and glucose. Finally, the labeling in the glucosyl and fructosyl moieties of sucrose were monitored by LC-MS/MS using the same parameters reported in Table 2, and the relative abundance of their mass isotopomers was determined (Table S2 (Supplementary Materials), Figure 4). The abundance of unlabeled intracellular sucrose (m_0_) quickly decreased to 15.7% in 1280 min, and then stabilized to approximately 10% towards the end of the incubation (Figure 4A,B). This profile is due to a fast appearance of m_6_ which reached 41.7% within 1280 min, and then plateaued at around 32%. In parallel, sucrose m_12_, m_9_, and m_3_ increased up to 14.0%, 10.3%, and 5.3%, respectively over the time course. The relative abundances of the unlabeled fructosyl and glucosyl moieties decreased at different rates (Figure 4C–F). Fructosyl m_0_ quickly dropped to 15.8% in 1280 min, whereas the abundance of glucosyl m_0_ was still at 55.2% at this time point and continued to slowly decline to 37.8% at 5600 min. For the m_6_ of sucrose fructosyl, the abundance rapidly culminated at 73.6% within 1280 min, and then diminished to 59.2% due to the appearance of other mass isotopomers, such as m_3_ that went up to 9.0% by the end of incubation. Fully labeled glucosyl moiety of sucrose constantly increased over the time course to reach 25.6% of abundance while the m_3_ plateaued to 12.3% after 2720 min.

With the methodology described here, the ^13^C-labeling was successfully determined in the fructosyl and glucosyl moieties of sucrose, which directly informs about the labeling of cytosolic F6P and G6P, respectively (Figure 1). The occurrence of m_3_ in sucrose fructosyl (F6P) is due to the reversibility of the aldolase, which combines two triose-phosphates into one hexose-phosphate (#3, Figure 1). Similarly, the appearance of m_3_ and m_6_ in sucrose glucosyl (G6P) reflects the activity of the phosphoglucose isomerase (#2, Figure 1). The fact that m_3_ increased in intracellular fructose demonstrates that a sucrose cycle of synthesis and degradation is occurring in developing maize embryos (#10 and 11, Figure 1). It is noteworthy to mention that there is no fructose 6-phosphatase indexed in MetaCyc nor KEGG Ligand database, so the only way to obtain fructose with three labeled carbons is through this sucrose substrate cycle. This is in accordance with previous ^13^C-metabolic flux analysis studies performed at isotopic and metabolic steady state [11,12,15,16,17,19]. Knowing that in the present work, maize embryos were fed with [U-^13^C]fructose and unlabeled glucose, the reactions to explain the incidence of labeling in intracellular glucose (m_3_ and m_6_) are by sucrose substrate cycle (#10 and 11, Figure 1) and/or a potential glucose 6-phosphatase activity (#9, Figure 1). The sucrose substrate cycle produces intracellular glucose and fructose at the same rate. However, the glucose m_3_ appeared 2.4 times faster than the fructose m_3_ (Figure 3A,C). This phenomenon underlines that both sucrose substrate cycle and glucose 6-phosphatase activity are simultaneously occurring in developing maize embryos. These results corroborate previous studies in maize root tips [13,14,20].

## 3. Materials and Methods

### 3.1. Chemicals

Glucose, fructose, glutamine, polyethylene glycol 4000 (PEG), 4-(2-Hydroxyethyl)piperazine-1-ethanesulfonic acid (HEPES), hexokinase (from *Saccharomyces cerevisiae*), invertase (from *Saccharomyces cerevisiae*), were purchased from Sigma (St. Louis, MO, USA). [U-^13^C] fructose was purchased from Isotec (Miamisburg, OH, USA). Acetonitrile, and methanol LC-MS grade were ordered from Fisher Scientific (Hampton, NH, USA).

### 3.2. Plant Materials and Growth Conditions

Maize (*Zea Mays* L.) seeds for ALEXHO S K SYNTHETIC C20 (Alex, NSL 117227) were obtained from the U.S. National Plant Germplasm System. Maize Alex plants were grown in a greenhouse, hand pollinated, and ears were harvested as previously described [16]. For cultures, 18 DAP maize ears were surface sterilized [26]. Briefly, after removing the inner husks and silks, maize ears were taken to a laminar flow bench. Each ear was sterilized for 20 min in 50% bleach (*v/v*), and then rinsed and soaked for 20 min in autoclaved water. Maize embryos were dissected under aseptic conditions, and transferred into the incubation media previously described [16,26]: glucose (200 mM), fructose (200 mM), glutamine (5 mM), MS basal salts (4.3 g/L), 15% polyethylene glycol 4000, MES buffer (10 mM), and a mixture of vitamins containing nicotinic acid (5 µg/mL), pyridoxine hydrochloride (0.5 µg/mL), thiamine hydrochloride (0.5 µg/mL), and folic acid (0.5 µg/mL). The pH was adjusted to 5.8 with 1N potassium hydroxide. Maize embryos were placed scutellum up in glass petri dishes, on double-glass fiber filters soaked with 8 mL of the medium described above. Petri dishes were sealed with surgical tape. For pulse labeling experiments, fructose was replaced by 100% [U-^13^C] fructose whereas glucose and glutamine remained unlabeled. Maize embryos were incubated in plates for 0, 1, 2, 5, 20, 80, 320, 1280, 2730, 4160, and 5600 min in the dark at 24 °C. One embryo was harvested for each time point as previously described [11]. Briefly, each embryo was collected and rinsed three times with 10 mL of water to remove surface labeling. The embryo was then frozen with liquid nitrogen and lyophilized for four days. Each pulse labeling experiment was conducted with four biological replicates.

### 3.3. Extraction of Soluble Sugars

Free soluble sugars were extracted from ground freeze-dried maize embryos using boiling water, and following an established procedure [9,24,26,27]. It is important to note that no 13C-labeled internal standards were added at the time of extraction. The extracts containing free sugars were freeze-dried, and stored at −20 °C until further analysis.

### 3.4. Analysis of 13C-Labeling in Free Sugars

An amount of 300 μL of ultra-pure water was added to the dried extract containing ^13^C-free intracellular sugars (fructose, glucose, and sucrose). ^13^C-sucrose was hydrolyzed into its ^13^C-fructosyl and ^13^C-glucosyl units, which are reflecting the labeling of cytosolic F6P and G6P, respectively. First, 100 μL of the free sugar sample were transferred into a 1.5-mL micro-centrifuge tube containing 500 μL of 100 mM HEPES (pH 7.6) buffer and 400 μL of ultra-pure water. 40 μL of this mixture were taken, mixed with 360 μL of ultrapure water, and added to a LC-MS glass vial containing 600 μL of 100% acetonitrile (control). Second, 10 μL of 1 M MgCl_2_, 10 μL of 100 mM ATP and 20 μL of 250 U/mL hexokinase prepared in HEPES (pH 7.6) were supplemented to the remaining 960 μL of the sample. Free ^13^C-fructose and ^13^C-glucose were: (i) phosphorylated for 2 h at 25°, (ii) placed in a boiling-water bath for 10 min in order to inactivate hexokinase, and (iii) directly transferred onto ice for 10 min. At this point, a 40 μL aliquot was taken and added to a 1.5 mL microcentrifuge tube containing 360 μL of ultrapure water. The 400 μL of extract was then transferred to a 3kDa Amicon filtering device, and spun at 14,000× *g* for 30 min. The eluate was added to a LC-MS glass vial containing 600 μL of 100% acetonitrile. Third, 40 μL of 1250 U/mL invertase were added to the sample followed by a 2 h incubation at 25 °C. After invertase treatment, a 400 μL aliquot was taken, loaded onto a 3 kDa Amicon filtering device, and centrifuged at 14,000× *g* for 30 min. Finally, the flow through was transferred into a LC-MS/MS glass vials containing 600 μL of 100% acetonitrile. The mass isotopomer distribution of free ^13^C-glucose, free ^13^C-fructose, ^13^C-glucosyl and ^13^C-fructosyl moieties generated from ^13^C-sucrose cleavage were analyzed by LC-MS/MS using multiple reaction monitoring mode. The LC and MS conditions were the same as those of previous work [24].

## Figures and Tables

**Table 1:** Mass spectrometry parameters for the detection of sugars. DP: Declustering potential, EP: Entrance potential, CE: Collision energy, CXP: Collision cell exit potential, are described for hexoses and disaccharides. Parent and daughter ion formulas were reported by Calvano et al. [25].

Sugars	Parent Ion Formula	Daughter Ion Formula	Parent/Daughter Transitions	DP (V)	EP (V)	CE (V)	CXP (V)
Hexoses	C_6_H_11_O_6_^-^	C_3_H_6_O_3_^-^	179/89	−30	−10	−12	−11
Disaccharides	C_12_H_21_O_11_^-^	C_2_H_4_O_2_^-^	341/59	−240	−10	−50	−9

**Table 2:** ^13^C-labeled sugar isotopomers using MRM. RT: Retention time.

Sugars	RT (min)	Mass Isotopomers	Parent/Daughter Transitions
Fructose	5.11	m_0_	179/89
Glucose	7.90	m_1_	180/89; 180/90
		m_2_	181/89; 181/90; 181/91
		m_3_	182/90; 182/91; 182/92
		m_4_	183/90; 183/91; 183/92
		m_5_	184/91; 184/92
		m_6_	185/92
Sucrose	11.95	m_0_	341/59
		m_1_	342/59; 342/60
		m_2_	343/59; 343/60; 343/61
		m_3_	344/59; 344/60; 344/61
		m_4_	345/59; 345/60; 345/61
		m_5_	346/59; 346/60; 346/61
		m_6_	347/59; 347/60; 347/61
		m_7_	348/59; 348/60; 348/61
		m_8_	349/59; 349/60; 349/61
		m_9_	350/59; 350/60; 350/61
		m_10_	351/59; 351/60; 351/61
		m_11_	352/60; 352/61
		m_12_	353/61

## Supplementary Materials

The following are available online at https://www.mdpi.com/2218-1989/10/1/30/s1, Table S1: ^13^C mass isotopomer abundances of glucose and fructose, Table S2: ^13^C mass isotopomer abundances of fructosyl and glucosyl moieties after sucrose cleavage.

## References

[1] Ratcliffe R.G., Shachar-Hill Y. (2006). Measuring multiple fluxes through plant metabolic networks. Plant J..

[2] Hatzfeld W.D., Stitt M. (1990). A study of the rate of recycling of triose phosphates in heterotrophic Chenopodium rubrum cells, potato tubers, and maize endosperm. Planta.

[3] Hill S.A., ap Rees T. (1993). Fluxes of carbohydrate metabolism in ripening bananas. Planta.

[4] Salon C., Raymond P., Pradet A. (1988). Quantification of carbon fluxes through the tricarboxylic acid cycle in early germinating lettuce embryos. J. Biol. Chem..

[5] Bowsher C.G., Tobin A.K. (2001). Compartmentation of metabolism within mitochondria and plastids. J. Exp. Bot..

[6] Lunn J.E. (2007). Compartmentation in plant metabolism. J. Exp. Bot..

[7] Dieuaide-Noubhani M., Alonso A.P. (2014). Application of metabolic flux analysis to plants. Methods Mol. Biol..

[8] Alonso A.P., Piasecki R.J., Wang Y., LaClair R.W., Shachar-Hill Y. (2010). Quantifying the Labeling and the Levels of Plant Cell Wall Precursors Using Ion Chromatography Tandem Mass Spectrometry. Plant Physiol..

[9] Cocuron J.C., Alonso A.P. (2014). Liquid chromatography tandem mass spectrometry for measuring ^13^C-labeling in intermediaries of the glycolysis and pentose-phosphate pathway. Methods Mol. Biol..

[10] Allen D.K., Shachar-Hill Y., Ohlrogge J.B. (2007). Compartment-specific labeling information in C-13 metabolic flux analysis of plants. Phytochemistry.

[11] Alonso A.P., Dale V.L., Shachar-Hill Y. (2010). Understanding fatty acid synthesis in developing maize embryos using metabolic flux analysis. Metab. Eng..

[12] Alonso A.P., Goffman F.D., Ohlrogge J.B., Shachar-Hill Y. (2007). Carbon conversion efficiency and central metabolic fluxes in developing sunflower (*Helianthus annuus* L.) embryos. Plant J..

[13] Alonso A.P., Raymond P., Hernould M., Rondeau-Mouro C., de Graaf A., Chourey P., Lahaye M., Shachar-Hill Y., Rolin D., Dieuaide-Noubhani M. (2007). A metabolic flux analysis to study the role of sucrose synthase in the regulation of the carbon partitioning in central metabolism in maize root tips. Metab. Eng..

[14] Alonso A.P., Raymond P., Rolin D., Dieuaide-Noubhani M. (2007). Substrate cycles in the central metabolism of maize root tips under hypoxia. Phytochemistry.

[15] Alonso A.P., Val D.L., Shachar-Hill Y. (2011). Central metabolic fluxes in the endosperm of developing maize seeds and their implications for metabolic engineering. Metab. Eng..

[16] Cocuron J.C., Koubaa M., Kimmelfield R., Ross Z., Alonso A.P. (2019). A combined metabolomics and fluxomics analysis identifies steps limiting synthesis in maize embryos. Plant Physiol..

[17] Dieuaidenoubhani M., Raffard G., Canioni P., Pradet A., Raymond P. (1995). Quantification of Compartmented Metabolic Fluxes in Maize Root-Tips Using Isotope Distribution from C-13-Labeled or C-14-Labeled Glucose. J. Biol. Chem..

[18] Masakapalli S.K., Le Lay P., Huddleston J.E., Pollock N.L., Kruger N.J., Ratcliffe R.G. (2010). Subcellular Flux Analysis of Central Metabolism in a Heterotrophic Arabidopsis Cell Suspension Using Steady-State Stable Isotope Labeling. Plant Physiol..

[19] Rontein D., Dieuaide-Noubhani M., Dufourc E.J., Raymond P., Rolin D. (2002). The metabolic architecture of plant cells - Stability of central metabolism and flexibility of anabolic pathways during the growth cycle of tomato cells. J. Biol. Chem..

[20] Alonso A.P., Vigeolas H., Raymond P., Rolin D., Dieuaide-Noubhani M. (2005). A new substrate cycle in plants. evidence for a high glucose-phosphate-to-glucose turnover from in vivo steady-state and pulse-labeling experiments with [^13^C] glucose and [^14^C] glucose. Plant Physiol..

[21] Fell D. (1997). Understanding the Control of Metabolism.

[22] Acket S., Degournay A., Merlier F., Thomasset B. (2017). 13C labeling analysis of sugars by high resolution-mass spectrometry for metabolic flux analysis. Anal. Biochem..

[23] Viola R., Davies H.V., Chudeck A.R. (1991). Pathways of starch and sucrose biosynthesis in developing tubers of potato (*Solanum tuberosum* L.) and seeds of faba bean (*Vicia faba* L.): Elucidation by ^13^C-nuclear-magnetic-resonance spectroscopy. Planta.

[24] Cocuron J.C., Anderson B., Boyd A., Alonso A.P. (2014). Targeted metabolomics of *Physaria fendleri*, an industrial crop producing hydroxy fatty acids. Plant Cell Physiol..

[25] Calvano C.D., Cataldi T.R.I., Kogel J.F., Monopoli A., Palmisano F., Sundermeyer J. (2017). Structural Characterization of Neutral Saccharides by Negative Ion MALDI Mass Spectrometry Using a Superbasic Proton Sponge as Deprotonating Matrix. J. Am. Soc. Mass Spectrom..

[26] Koubaa M., Cocuron J.-C., Thomasset B., Alonso A.P. (2013). Highlighting the tricarboxilic acid cycle: Liquid and gas chromatography-mass spectrometry analyses of ^13^C-labeled organic acids. Anal. Biochem..

[27] Cocuron J.C., Tsogtbaatar E., Alonso A.P. (2017). High-throughput quantification of the levels and labeling abundance of free amino acids by liquid chromatography tandem mass spectrometry. J. Chromatogr. A.

